# Continuity or change? How the onset of COVID‐19 affected internal migration in Australia

**DOI:** 10.1002/psp.2626

**Published:** 2022-10-27

**Authors:** Francisco Perales, Aude Bernard

**Affiliations:** ^1^ School of Social Science The University of Queensland Brisbane Queensland Australia; ^2^ School of Earth and Environmental Sciences The University of Queensland Brisbane Queensland Australia

**Keywords:** Australia, COVID‐19, HILDA, internal migration, mobility, pandemic, reasons for migrating, residential mobility, residential relocation, trends

## Abstract

Despite anecdotal evidence of a COVID‐19 induced decline in the intensity of interstate migration in Australia, population‐level evidence is limited. The recent release of the 2020 wave of the Household, Income and Labour Dynamics in Australia (HILDA) Survey provides a unique opportunity to robustly assess the effect of the COVID‐19 pandemic on the level, direction, determinants, and reasons for migration in Australia. By applying a series of regression models to individual‐level longitudinal microdata, and measuring migration at a range of spatial scales, this paper shows that COVID‐19 has somewhat accelerated the long‐term decline in the intensity of internal migration—particularly for residential mobility, short‐distance migration, and migration due to employment and involuntary reasons. The socio‐demographic determinants of migration have remained broadly stable, despite a slight increase in the deterring effect of duration of residence and a reduction in the impact of education. Finally, we show that the increase in net migration gains in regional areas is underpinned by a decrease in outflows. Juxtaposing these results with aggregate‐level migration statistics from the Australian Bureau of Statistics from 2021, we conclude that the effect of COVID‐19 on internal migration to date has been minimal and is likely to be short‐lived. However, it may still be too soon to make a definitive judgement, as shifts in work patterns stemming from the pandemic may further transform the level, direction, and composition of internal migration.

## 1 INTRODUCTION

While recent studies have focused on the effect of COVID‐19 on mortality (Morosow & Kolk, 2020), interest in the demographic consequences of the pandemic is progressively shifting to encompass other drivers of population change, including fertility (Berrington et al., 2022) and migration (Anderson et al., 2021). The emerging body of evidence suggests that the demographic effects of COVID‐19 will be far‐reaching. For example, in many high‐income countries, the COVID‐19 pandemic is expected to result in record low fertility (Berrington et al., 2022; Fostik, 2021; Luppi et al., 2020). This is the case in Australia, where the present study is based, which recorded its lowest total fertility rate on record in 2020 at just 1.58 (ABS, 2021). By affecting the size of new birth cohorts (Aassve et al., 2020), this decline in fertility is likely to extend the demographic reach of COVID‐19 for generations. The effect of declining fertility rates has been compounded by the closure of national borders, which brought international migration to halt in many countries (Gamlen, 2020; Newland, 2020). This includes OECD countries, where permanent migration flows fell by 30% in 2020 (OECD, 2021). Despite a progressive easing of restrictions, international migration is yet to return to prepandemic levels (IOM, 2022). For a high‐immigration country such as Australia, which remained closed to nonresidents for nearly 2 years, the flow‐on effects are expected to be significant. The Australian population is forecast to be 1.9 million smaller by 2040 than it would have been in the absence of COVID‐19 (Charles‐Edwards et al., 2021) because of a million fewer immigrants and close to 800,000 fewer births over the next 20 years.

There have been comparatively fewer studies on the impact of COVID‐19 on internal migration, despite the importance of internal migration in redistributing population within countries (Rees et al., 2017) and the fact that, globally, internal migrants outnumber international migrants by a factor of 4 to 1 (Bell & Muhidin, 2009). While limited, evidence emerging from developed countries suggests three key COVID‐19‐related changes in internal migration: (i) a decrease in the level of internal migration, particularly among young adults (Stawarz et al., 2022); (ii) a change in the direction of flows in favour of regional areas (González‐Leonardo et al., 2022), especially among higher income earners (Haslag & Weagley, 2021); and (iii) a decrease in the incidence of employment‐related migration accompanied by a growth in lifestyle migration (Haslag & Weagley, 2021). However, the extent of these shifts appears to be country specific. For example, Germany recorded a 5% decline in inter‐county migration between 2019 and 2020 (Stawarz et al., 2022), compared to 2.5% in Spain (González‐Leonardo et al., 2022). More importantly, there seem to be inconsistencies in the persistence of these shifts. Spain has already returned to prepandemic patterns of internal migration (González‐Leonardo et al., 2022), while the United States is yet to see a reversal in the net flows out of cities (Haslag & Weagley, 2021).

To shed further light on the impact of COVID‐19 on internal migration, the present study extends the evidence to Australia, one of the most mobile countries in the world (Bell et al., 2015) despite a sustained decline in the level of internal migration since the 1990s (Bell et al., 2018; Kalemba et al., 2020). The Australian migration system has long been characterised by net population losses for the state of New South Wales, with compensating gains in the state of Queensland, which has been the main migration destination since the 1970s. At a substate level, there has been a long‐standing pattern of net losses from metropolitan areas in favour of outer suburbs (Ford, 1999), coupled with a migration stream to high‐amenity regional areas and rural towns (Argent et al., 2010). Following the outbreak of the COVID‐19 pandemic, Australia closed its international borders to nonresidents in March 2020. Various restrictions to interstate travel were then introduced ranging from bans on nonessential travel to New South Wales and Victoria to the closure of state borders to nonresidents in Queensland and Western Australia. Stricter local restrictions were put in place in the subsequent COVID‐19 waves that affected Melbourne from June to October 2020 and New South Wales and Victoria from August to October 2021, bringing movement within those states to a halt. In other states, temporary border closures and snap lockdowns were implemented in response to localised outbreaks, such as in Brisbane in March 2021. State and international borders progressively reopened from December 2021 onward, with Western Australia being the last state to fully re‐open its borders with the rest of Australia in April 2022.

Evidence on the impact of COVID‐19 in Australia remains limited because of the absence of cotemporaneous micro‐data. Studies to date are descriptive and based on aggregate flows at coarse levels of geography (Borsellino et al., 2022; Wilson & Grossman, 2021). More concerning is the suspension by the Australian Bureau of Statistics (ABS) of its quarterly release of internal migration data from mid‐2021 onwards. This is because intercensal migration estimates are derived from data from Medicare, Australia's universal medical insurance scheme, which has recorded an unprecedented spike in changes of address because of Australia's COVID‐19 mass vaccination programme. In this context, this paper draws on longitudinal microdata from the Household, Income and Labour Dynamics in Australia (HILDA) Survey, whose latest wave was fielded in mid‐2020 and released in late 2021. This nationally representative survey permits: (i) extending understandings of the impact of the COVID‐19 pandemic on internal migration to lower levels of geography; (ii) progressing analyses beyond descriptive statistics by using a regression framework to control for personal characteristics and identifying possible changes in the socio‐demographic profile of internal migrants; and (iii) expanding the analysis to the realisation of migration intentions and reasons for migrating.

The remainder of the paper begins by reviewing the existing evidence on the impact of COVID‐19 on internal migration in developed countries. Section 3 introduces the data and presents our methods based on three complementary regression‐based tests to ascertain whether the onset of COVID‐19 pandemic affected the (i) intensity of internal migration, (ii) reasons for migrating, (iii) direction of migration flows between capital cities and regional areas, (iv) realisation of migration expectations and (v) determinants of migration. Results are presented in Section 4. Section 5 concludes by summarising post‐COVID‐19 patterns of internal migration and implications for future research.

## 2 COVID‐19 AND INTERNAL MIGRATION IN DEVELOPED COUNTRIES

To understand the impact of the COVID‐19 outbreak on internal migration, it is important to distinguish between three distinct but interrelated dimensions of migration: its level or intensity; its spatial structure, and its selectivity and flow composition. In this section, we review each of these dimensions. We both outline theoretical explanations for changes in each dimension and synthesise emerging evidence on the impact of COVID‐19 on internal migration in developed countries. For Australia, we analyse recently released aggregate migration estimates from the Australian Bureau of Statistics capturing migration between states, as well as between capital cities and regional areas.

### 2.1 Intensity of migration

The intensity of internal migration—that is, the proportion of the population changing region of residence each year—is known to vary with the business cycle, as prospective migrants respond to opportunities (or lack thereof) in the housing and labour markets. The intensity of migration has been shown to trend up in expansionary phases of the business cycle and to go down when the economy contracts. This pattern has been observed in many OECD countries (Alvarez et al., 2021; Van Der Gaag & Van Wissen, 2008), although the effects of the Great Recession of 2007–2009 on internal migration were more pronounced in some countries such as the United States (Monras, 2018) than others such as Italy (Bonifazi et al., 2020). The enduring relationship between internal migration and macro‐level economic conditions suggests that any economic slowdown caused by COVID‐19 is likely to have caused a fall in internal migration. The dampening effect of worsening economic conditions on internal migration is likely to have been exacerbated by the lockdowns of 2020 and early 2021 because of restrictions on movement to some states.

**Figure 1:**
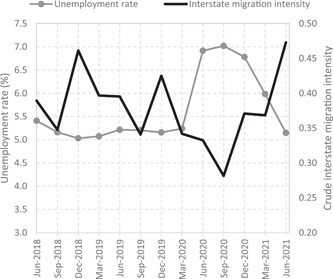
Quarterly unemployment rate and crude interstate migration intensity, from June 2018 to 2021, Australia. Sourced from the Australian Bureau of Statistics, Labour force, seasonally adjusted unemployment rate (6202.0) and National, state and territory population (3101.0).

Pioneer evidence from developed countries confirms a decline in the intensity of internal migration post‐COVID‐19. Germany recorded a 5% decrease in the intensity of inter‐county migration between 2019 and 2020 (Stawarz et al., 2022). In Spain, the decline in internal migration has been more limited (2.5%) and short‐lived, with a return to prepandemic levels by the end of 2020 (González‐Leonardo et al., 2022). Quarterly data for Australia shown in Figure 1 reveals a decline in the intensity of interstate migration between December 2019 and September 2020, followed by a rebound. The highest quarter‐on‐quarter decreases were recorded between December 2019 and March 2020 (−19.55%) and between June and September 2020 (−15.39%). This trend mirrors changes in the unemployment rate, which spiked from June 2020 to March 2021 before a return to prepandemic levels. As a result, the interstate migration intensity in June 2021 was higher than in June 2020, 2019 and 2018, which suggests that any impacts of COVID‐19 in Australia may have been short‐lived.

**Figure 2:**
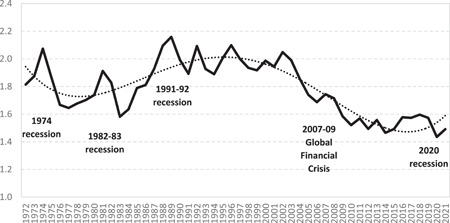
Crude interstate migration rate, from 1978 to 2020, Australia. Sourced from Australian Bureau of Statistics, Australian Historical Population Statistics for 1971–2019 and Regional Populations for 2020 and 2021. The trend line is a fourth‐degree polynomial represented by a dotted line.

Because quarterly estimates are sensitive to seasonal variations, we also report annual data in Figure 2. This shows an 8.75% decline in the intensity of interstate migration between 2019 and 2020, resulting in the lowest annual level of interstate migration on record at 1.44%. However, this is not the largest year‐on‐year decrease. The largest decrease was in fact recorded during the recession of the early 1980s, where migration dropped by 13.51% in a year. In addition, the COVID‐19‐induced decline seems to have been short‐lived compared to the decline during earlier recessions, when interstate migration went down for at least 2 consecutive years. Indeed, the 2020 decrease in interstate migration was followed by a 3.88% rebound in 2021. Using an econometric model based on past relationships between interstate migration and economic variables, Bernard et al. (2020) forecast interstate migration in Australia to ‘bottom out’ during the 2020–2021 financial year, before bouncing back in the next 2 years and stabilising at 2018–2019 levels. This trend is also forecast by the Australian Commonwealth Department of Treasury in its 2022–2023 budget (CPoP, 2022).

In the absence of contemporaneous data, these results collectively suggest that the decline in interstate migration that followed the onset of COVID‐19 is likely to be temporary. However, in contrast with other countries, there is no evidence to date in Australia on the impact of COVID‐19 on short‐distance migration, including intrastate migration and residential mobility.

### 2.2 Spatial structure of migration

There is growing evidence that the fall in the intensity of migration post‐COVID has been accompanied by minor changes in the direction of flows in favour of nonmetropolitan regions. In Germany, for instance, this has been manifested by an increase in net losses for the largest cities caused by lower inflows of young adults combined with sustained outflows of families (Stawarz et al., 2022). Similarly, in Japan, Tokyo recorded net population losses due to a reduction in in‐migration flows (Fielding & Ishikawa, 2021). In the United States, a similar pattern was observed. Between April 2020 and December 2021, there is evidence of flows out of the more populous cities in favour or smaller cities (Haslag & Weagley, 2021). In Spain, large cities such as Barcelona and Madrid recorded net population losses in 2020 because of an increase in out‐migration coupled with a decrease in in‐migration. Conversely, rural areas—particularly tourist destinations and coastal and mountain areas close to cities and natural parks—gained population due to internal migration. However, the largest flows continued to be from core cities to suburbs and between urban areas. Importantly, by the end of 2020, internal migration in Spain had returned to prepandemic patterns (González‐Leonardo et al., 2022).

In Australia, the decline in the intensity of internal migration post‐COVID‐19 has also been overlaid by a minor change in the spatial structure of migration. This involves a growth in net losses for Sydney and Melbourne and corresponding net gains for regional New South Wales and Victoria (Wilson & Grossman, 2021). While Queensland has recorded a stable net migration rate for its capital city (Brisbane) and a growth for its regional areas, the absence of origin–destination matrix data has not permitted the establishment of the sources of these population gains. However, a recent analysis of in and out‐flows suggests that the increase in regional net population gain is the combined result of increases in people staying in regions and leaving cities (Borsellino et al., 2022). This can be seen in Figure 3, which plots in, out, and net migration rates for greater capital cities and rest of state from 2018–2019 to 2020–2021. The figure shows that net migration losses for Melbourne and Hobart increased post‐COVID. Brisbane recorded sustained net migration gains, and Perth and the Australian Capital Territory turned from negative to positive net migration rates, while Adelaide and Darwin recorded a decrease in net losses. Most notably, in all states, the growth in net gains for regional areas, or the reduction in net losses in the case of Western Australia and the Northern Territory, have been mainly driven by a decrease in out‐migration. However, the magnitude of these shifts is only moderate, and the gains for regions remain minimal. Hence, it is clear from Figure 3 that COVID‐19 has not led to an overhaul of the internal‐migration system in Australia.

Figure 3In, out and net migration rates for greater capital cities and rest of state from 2018–2019 to 2020–2021. Sourced from Australian Bureau of Statistics, Regional population.

**Figure:**
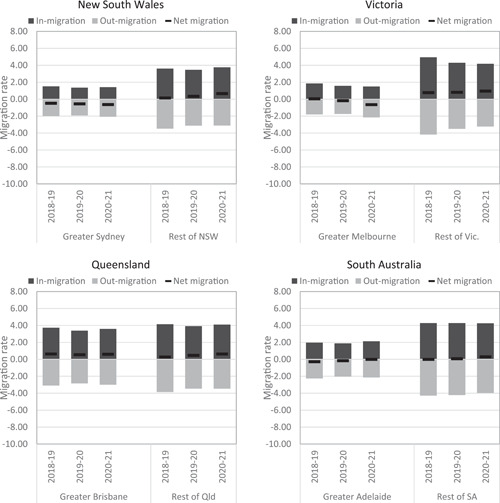

**Figure:**
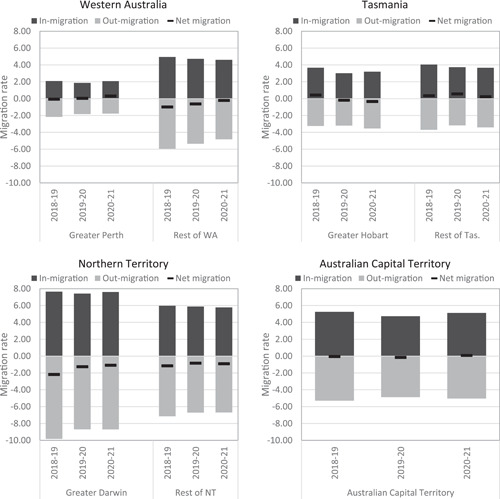

These shifts have been tentatively explained by the growth in teleworking, stricter lowdown in large cities, particularly Sydney and Melbourne, which were the epicentre of COVID‐19 infections and mortality. In the absence of contemporaneous data due to the suspension of the ABS's regional migration estimates, geographers have turned to forecasting. Using an Autoregressive Integrated Moving Average (ARIMA) model, Borsellino et al. (2022) argued that net gains to regions will slow down after 2022, but remain higher than prepandemic levels in New South Wales, Queensland, South Australia, and Tasmania. It is worth nothing that net migration gains for regional areas of New South Wales, Victoria, Queensland and Tasmania predate the pandemic and are part of a longer‐term trend underpinned by affordable and attractive lifestyles in regional areas (Argent & Plummer, 2022).

### 2.3 Selectivity of migration

An important question for population geographers and demographers is whether changes in migration behaviour are uniform across the population. Migration is well‐established to be selective, particularly with respect to age (Bernard et al., 2014; Rogers & Castro, 1981). Evidence from Germany suggests that the COVID‐19‐induced decline in internal migration has been more pronounced among young adults (Stawarz et al., 2022). However, in Australia, the beginning of the pandemic prompted many young adults to return to their parental home. An estimated 268,000 adult children returned to the parental home by May 2020 (ABS, 2020) and close to 4% of Australian households hosted someone temporarily because of the pandemic, of whom over 37% were hosting their adult children. Return migration is likely to have resulted in flows in the reverse direction after restrictions eased. More importantly, age differentials appear to be overlaid by socioeconomic variation. In the United States, migration out of cities has been predominantly observed among higher‐income earners and white‐collar workers who are more likely to work remotely (Bick et al., 2021). This is reflected in a shift in reasons for migrating in favour of nonwork‐related reasons in the United States (Haslag & Weagley, 2021).

**Figure 4:**
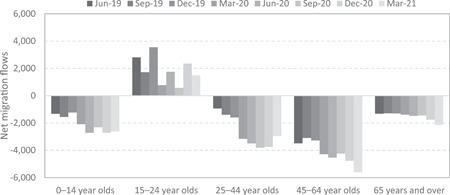
Quarterly net migration flows to capital cities by age groups. Authors' calculations based on provisional regional internal migration estimates from the Australian Bureau of Statistics. This quarterly time series was created in response to the COVID‐19 pandemic and estimates for earlier periods are not available.

**Figure 5:**
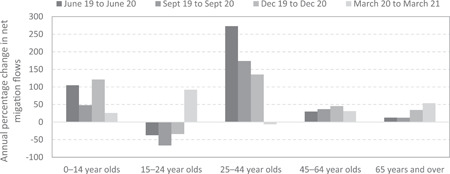
Annual percentage change in net migration flows to capital cities areas by age groups. Authors' calculations based on provisional regional internal migration estimates from the Australian Bureau of Statistics.

Figure 4 reports quarterly net migration flows between capital cities by age group from June 2019 to March 2021 for Australia. It clearly shows a growth in net losses in the key working age groups of 25–64 years old, coupled with a decline in net gains of young adults aged 15–24.^1^ To control for seasonal variations, Figure 5 reports the annual percentage change in net migration flows to capital cities, showing that changes in migration patterns were highest for the 24–44 age group. However, the age patterns of net losses and gains have stabilised. Net losses for the 25–44 age group increased by over 270% from June 2019 to 2020, but only by 135% in 12 months to December 2020. More importantly, net losses for the 25–44 age group reduced by 7% between March 2020 and 2021. This trend suggests that flows between capital cities and regional areas are progressively returning to prepandemic levels. However, there is no Australian evidence to date on the impact of COVID‐19 on other socio‐demographic determinants of internal migration. This study aims to shed new light on early disruptions to internal migration trends and patterns in Australia following the onset of the COVID‐19 pandemic.

## 3 DATA AND METHODS

### 3.1 The HILDA Survey

To accomplish our analytic aims, we leverage 20 years of panel data from the HILDA Survey (Watson & Wooden, 2012). HILDA is a high‐quality, multipurpose, panel survey that has interviewed individuals living in the same households between 2001 and 2020. It features a complex probabilistic sampling design that makes it representative of the Australian population (Summerfield et al., 2021). Further, the survey has remarkably high wave‐on‐wave retention rates by international standards (~90%–95% or respondents per wave), and panel attrition has had a limited impact on aggregate‐level measures of internal migration (Watson, 2020). Further, trends in intra‐ and inter‐state migration in the HILDA Survey have been shown to be comparable to those recorded at successive Australian population censuses (Kalemba et al., 2022), despite minor differences due to nonresponse and recall errors in the census, particularly among older people (Watson, 2020).

The HILDA Survey is an ideal data set to comprehensively assess the effect of COVID‐19 on internal migration, as it allows tracking internal migration at a range of spatial scales before and after the onset of the pandemic. It also has the unique advantage of collecting data on migration reasons and intentions, as well as rich longitudinal information on respondents' characteristics that may confound the relationships of interest. The HILDA Survey has been used extensively in earlier Australian migration studies encompassing a broad range of topics, including the determinants of migration (Campbell, 2019; Crown et al., 2020), its social and economic impacts (Clark & Lisowski, 2019; Korpi & Clark, 2017), and its association with life‐course transitions (Bernard et al., 2016; Clark & Lisowski, 2018; Sander & Bell, 2014; Vidal et al., 2017). However, these studies precede the onset of COVID‐19.

HILDA survey interviews typically take place between August and February, which means that the 2020 data have been collected approximately 5 to 11 months after the pandemic's onset. This offers a unique window into its possible early effects on internal‐migration trends and patterns. Our analyses are based on HILDA Survey data stretching from 2003 to 2020. The 2001 data cannot be incorporated into the analysis because there is no suitable information about the respondents' area of residence before entering the panel. For the same reason, we also excluded the first observation from all new entrants (e.g., those joining participating households, turning 15 years of age while living in a participating household, or who are part of the 2011 booster sample). The 2002 data were not used due to coding issues in the identification of statistical areas.^2^ To maximise statistical power, we use all observations with nonmissing data on the control and outcome variables. The resulting data set has an unbalanced panel design and sample sizes differ slightly across models using different outcome measures (from 252,870 to 252,954 observations).

### 3.2 Migration variables

We measure migration by comparing respondents' place of residence at times *t* and *t* − 1. We do so at a range of spatial scales based on the Australian Statistical Geographic Standard (ASGS) to examine whether the pattern of results is consistent across different types of moves. The ASGS is a social‐geography classification developed to reflect the location of people and communities that classifies Australia into a nested hierarchy of statistical areas. Areas range from SA1s (the smallest units) to states and territories (the largest units), as shown in Table A1 in the Appendix. We complement the ASGS‐based measures with a commonly used measure of change of address (Courgeau et al., 2012; Long, 1991) and a measure of changes across Greater Capital City Statistical Areas (GCCSA), which captures migration between capital cities and rest of states. For each geography, we calculate annual crude migration intensities^3^ (CMIs), defined as the number of people who migrated during a year divided by the population (i.e., HILDA's total annual sample), expressed as a percentage for descriptive statistics.

We also measure migration intensities based on self‐reported reasons or reason‐specific migration intensities, for all changes of address, irrespective of the spatial scale. We choose this measure because it allows us to overcome the problem of small sample size. Despite the limitations of self‐reported migration reasons, including ex‐post rationalisation (Gillespie et al., 2021), such data offer unique and valuable insights into the motives underpinning the decision to move (Thomas et al., 2019). We group reasons for migrating into six categories, as done by Kalemba et al. (2022): (i) *employment reasons* (start a new job, be nearer to a place of work/study, work transfer, start or relocate one's business, look for work, other work reason); (ii) *housing reasons* (get a larger, better, smaller, cheaper or own place); (iii) *amenity reasons* (live in a better neighbourhood, be closer to amenities/services, or for another housing/neighbourhood reason); (iv) *family reasons* (moving in with a partner, be closer to friends/family, follow family members, relationship breakdown, other family reason); (v) *involuntary reasons* (property no longer available, eviction, or government housing restrictions); and (vi) *lifestyle reasons* (lifestyle change, health reasons).

Using the GCCSA measure introduced above, we also calculate migration intensities between regional and urban areas to assess whether their magnitude and direction have changed since the onset of COVID‐19. We distinguish between people who: (i) stayed in the same capital city; (ii) moved interstate across capital cities; (iii) stayed in the same regional area; (iv) moved interstate to regional areas; (v) moved from a capital city to a regional area; and (vi) moved from a regional area to a capital city. In descriptive statistics, we report them as a percentage of the population.

To examine the realisation of migration intentions, we derive a variable capturing the (mis)match between individuals' self‐reported likelihood to move over the next 12 months (time *t* − 1) and their subsequent address changes (time *t*; Coulter et al., 2011). Respondents were deemed to report being likely to move if they chose the responses ‘likely’ or ‘very likely’ out of a 5‐point Likert scale. The resulting variable had the following categories: (i) expected to move and did move; (ii) expected to move, but did not move; (iii) did not expect to move, but moved; and did not expect to move and did not move. Again, in descriptive analyses, these are reported as a percentage of the population.

#### 3.2.1 Control variables

The control variables in our multivariable models described below include a range of socio‐demographic and economic factors known to be drivers of internal migration. These include categorical variables capturing respondents' age group (14–24 years; 25–44 years; 45–64 years; 65+ years), gender (female; male), immigrant status (Australian born; not Australian born), partnership status (married/cohabitating; divorced, separated, or widowed; never married), parenthood status (not parent; parent), educational qualifications (no university degree; university degree), labour‐force status (employed full‐time; employed part‐time; unemployed; not in the labour force), household financial year disposable regular income (bottom quartile; middle two quartiles; top quartile); housing tenure (owns home; rents; other arrangement), duration of residence in current address (less than 1 year; 1–5 years; 5–10 years; 10+ years; insufficient information to determine) and state‐of‐residence fixed effects. Descriptive statistics for all variables, pooling all time periods, are presented in Table A2 in the Appendix.

### 3.3 Analytic strategy

We complement descriptive statistics with regression analyses to rule out that changes in internal migration post‐COVID‐19 are due to underlying changes in the composition of the population. We conduct three complementary analytic tests to assess whether the COVID‐19 pandemic affected the intensity of internal migration, reasons for migrating, direction of migration flows between capital cities and regional areas, and realisation of migration expectations. These three tests involve estimating whether a given internal‐migration outcome is significantly different in 2020 compared to 2019 (Test 1); whether the change in that migration outcome occurring between 2020 and 2019 is significantly different to the change occurring between 2019 and 2018 (Test 2); and whether the level of that internal‐migration outcome in 2020 deviates significantly from the long‐term trajectory since 2003 (Test 3). Each approach has its advantages and disadvantages relative to the others. Hence, we triangulate information from all three tests to robustly assess the effect of COVID‐19 on our outcomes of interest.

For each migration measure, Tests 1 and 2 are implemented through the following logistic regression model:

(Model 1) $$\log\left(\frac{\text{Pr}(M=1)}{1\text{‐Pr}(M=1)}\right)=\text{α}+\text{β}Y_{2003}+\text{β}Y_{2004}+\text{…}+\text{β}Y_{2020}+\delta X+e$$

Here, α is the model's intercept; M is a binary variable representing one migration measure; Y_2003_ to Y_2020_ is a set of dummy variables capturing survey year (with year 2019 as the reference category) and the βs are the associated model coefficients; X is the set of control variables and δ the associated vector of model coefficients; and e is an idiosyncratic error term. Model coefficients are expressed as odds ratios (ORs). The key parameter for Test 1 is the coefficient on Y_2020_, which compares mobility rates in 2020 and 2019. If the OR on βY_2020_ is smaller than one and its (two‐sided) *p* value is smaller than 0.05, then we would conclude that the mobility rate is significantly smaller in 2020 than in 2019. Test 2 is implemented postestimation, as a Wald test of the following form: (*βY*_*2020*_ − *βY*_*2019*_) − (*βY*_*2019*_ − *βY*_*2018*_) < 0. If this expression holds and its (one‐sided) *p* value is smaller than 0.05, then we would conclude that any decrease in the mobility rate between 2020 and 2019 is significantly larger than any decrease between 2019 and 2018.

To implement Test 3, we fit a second logistic regression model for each mobility outcome taking the following form:

(Model 2) $$\log\left(\frac{\text{Pr}(M=1)}{1\text{‐Pr}(M=1)}\right)=\text{α}+\text{β}\text{Year}+\text{β}Y_{2020}+\delta X+\text{e}$$

The main difference between Model 1 and Model 2 is the specification of the time variables. Here, the survey year is captured by a continuous variable going from 2003 to 2020 (βYear, the linear trend) and a dummy variable for year 2020 (β_2020_, the ‘COVID disruption’). The OR on β_2020_ constitutes a test of whether the mobility rate observed in 2020 deviates significantly from the (linear) long‐time trend. If the OR is greater than one and its (two‐sided) *p* value is smaller than 0.05, then we would conclude that there is significant disruption from the long‐term trends in 2020.

When analysing migration reasons, each reason is modelled independently. This is because the different reasons are not mutually exclusive, with ~16% of movers reporting more than one. When modelling the two multi‐categorical outcome variables (i.e., the direction of migration flows between capital cities and regional areas and the realisation of migration expectation), we use multinomial logistic regression instead of binary logistic regression. These results are reported as relative risk ratios (RRRs).

We conclude the analysis by exploring possible changes in the socio‐demographic factors that facilitate or constrain migrations. To that end, we fit a series of logistic regression models—for each spatial scale—using HILDA Survey data from years 2019 and 2020. In these models, we interact each of the explanatory variables with a dummy variable for year 2020. The resulting interaction effects give the (multiplicative) change in odds of migrating in 2020 relative to 2019 for a given explanatory variable.

## 4 EMPIRICAL EVIDENCE

### 4.1 Internal migration trends and COVID‐19: Descriptive statistics

**Figure 6:**
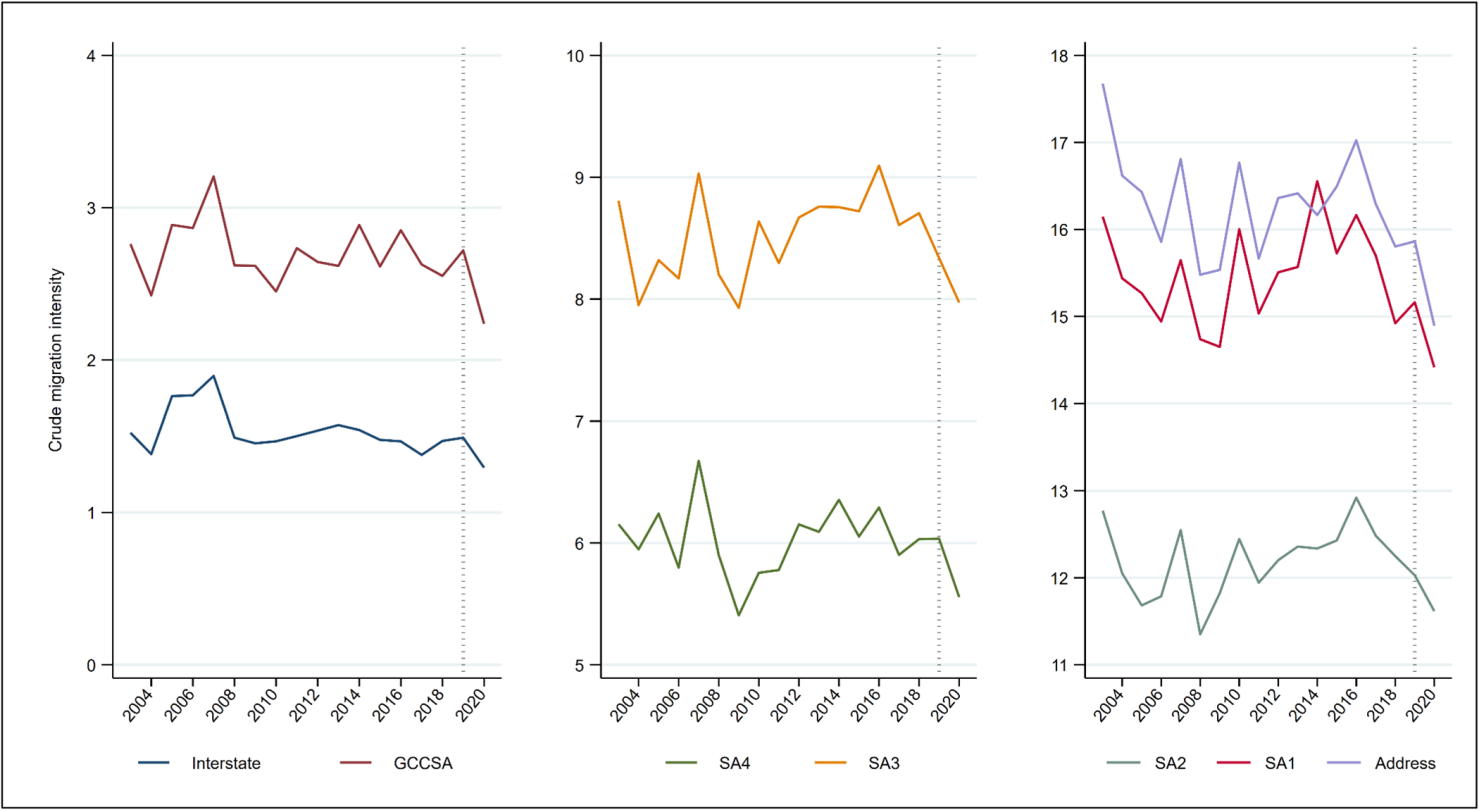
Crude migration intensity, at various spatial scales, from 2003 to 2020. Data from the HILDA Survey, from 2003 to 2020. GCCSA, Greater Capital City Statistical Area; SA, Statistical Area.

**Figure 7:**
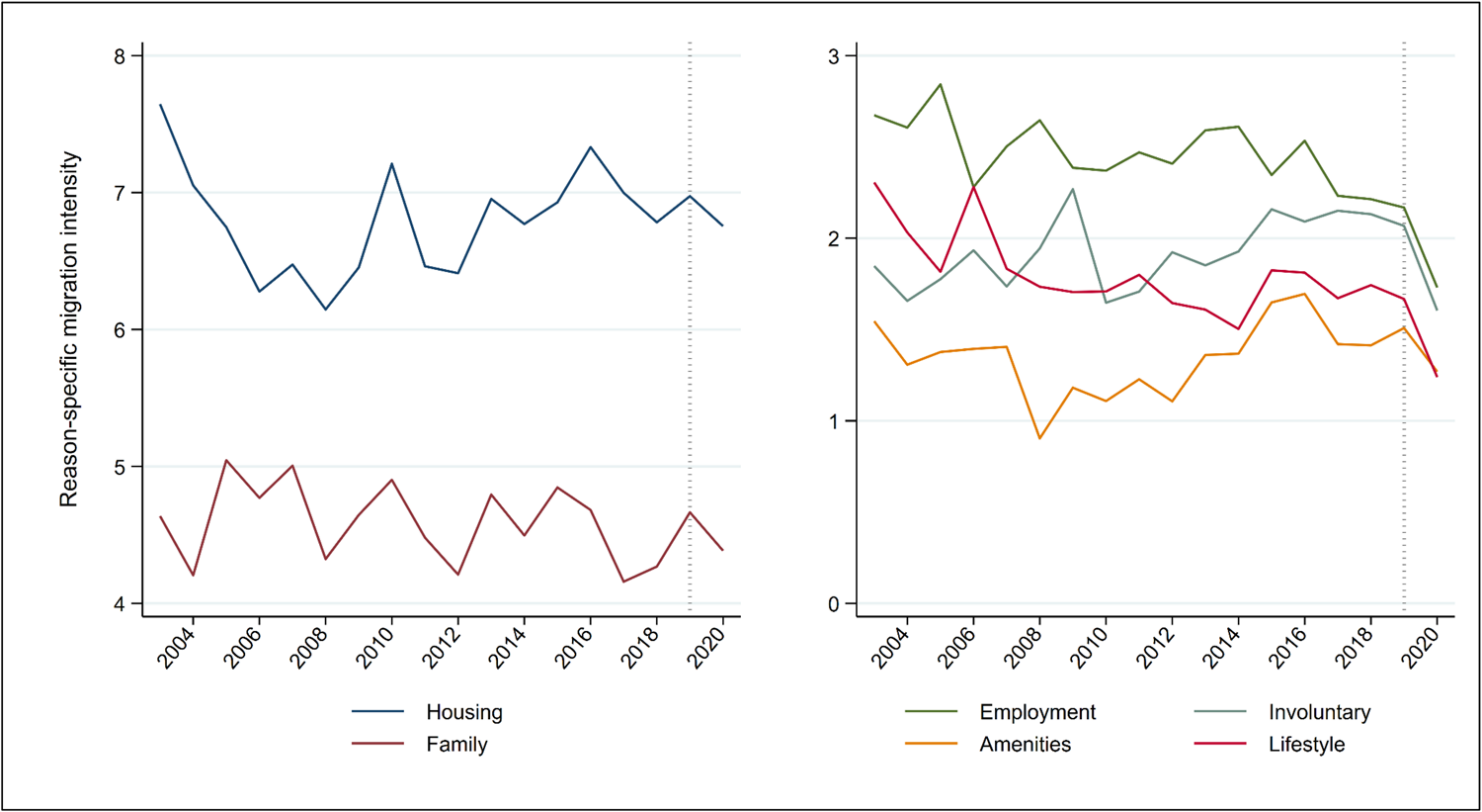
Reason‐specific migration intensity, from 2003 to 2020. Data from the HILDA Survey, from 2003 to 2020. HILDA, Household, Income and Labour Dynamics in Australia.

Figures 6 and 7 show annual crude migration intensities and reason‐specific migration intensities in Australia over the 2003–2020 period. The right‐hand side of the chart, marked by a vertical dashed line, represents the onset of the COVID‐19 pandemic. Figure 6, focusing on crude migration intensity at different spatial scales, portrays an overall picture of decline in internal migration at all spatial scales, but a modest decline. Between 2019 and 2020, the intensity of migration dropped for address changes (from 15.9% to 14.9%) and changes across SA1s (15.2%–14.4%), SA2s (12.0%–11.6%), SA3s (8.3%–8.0%), SA4s (6%–5.5%), GCCSAs (2.7%–2.2%) and states/territories (1.5%–1.3%). These declines, however, appear of comparable magnitude to those observed for some of the earlier years, including those for interstate and inter‐GCCSA migration following the 2007–2009 Global Financial crisis.

Figure 7, focusing on reasons for migrating, tells a similar story. Between 2019 and 2020, the share of the population migration for each reason dropped. This includes moves motivated by employment reasons (falling from 2.2% to 1.7%), housing reasons (6.5%–6.3%), amenities reasons (1.5%–1.3%), family reasons (4.2%–3.9%), involuntary reasons (2.1%–1.6%) and lifestyle reasons (1.7%–1.2%). Again, while the analysis portrays a consistent picture of decreasing migration, the declines are not dissimilar in magnitude to those observed for earlier time periods. In terms of the percentage changes, the decline was greatest for employment, lifestyle and involuntary migration, resulting in employment and lifestyle migration being the lowest on record. In the next section, we use multivariable modelling to formally test whether the declines shown in Figures 6 and 7 can be taken as evidence of a ‘COVID disruption’ to long‐ and short‐term trends in migration in Australia.

### 4.2 Testing for COVID disruptions to migration trends

Tables 1 and 2 show the results of our logistic regression models of different migration outcomes. As explained before, all models are adjusted for year‐on‐year differences in key socio‐demographic and economic factors. Table 1 reports the results for migration measured at different spatial scales, whereas Table 2 focuses on reasons for migrating. The top panel in each table contains the results of our first regression specification, comparing the 2020 mobility rates to the 2019 rates, as well as the magnitude of the change between 2020 versus 2019 and that between 2019 versus 2018. The bottom panel in each table contains the results of our second specification, which estimates whether year 2020 falls outside of a long‐term linear mobility trend.

**Table 1:** Odds ratios (*p* values) from logistic regression models of migration at different spatial scales

	Changed address	Changed SA1	Changed SA2	Changed SA3	Changed SA4	Changed GCCSA	Changed state
*Panel 1 (Model 1)*							
Year 2018	0.99	0.97	1.02	1.05	1.00	0.92	0.96
	(0.77)	(0.40)	(0.58)	(0.23)	(0.98)	(0.26)	(0.68)
Year 2019 *(reference)*							
**Test 1**: Year 2020	0.92*	0.93*	0.95	0.95	0.91#	0.81**	0.86
	(0.02)	(0.04)	(0.18)	(0.22)	(0.06)	(<0.01)	(0.11)
**Test 2**: one‐sided *p* value	0.06	0.04	0.32	0.49	0.14	0.01	0.12
*n* (observations)	252,954	252,912	252,912	252,912	252,912	252,941	252,954
*n* (individuals)	26,717	26,715	26,715	26,715	26,715	26,717	26,717
Pseudo *R* ^2^	0.161	0.166	0.150	0.127	0.106	0.082	0.094
*Panel 2 (Model 2)*							
Year (continuous)	0.99**	0.99**	1.00**	1.00*	0.99**	0.99*	0.99**
	(<0.01)	(<0.01)	(<0.01)	(0.05)	(<0.01)	(0.01)	(<0.01)
**Test 3**: Year 2020	0.94*	0.93*	0.95#	0.93*	0.93#	0.86*	0.92
	(0.03)	(0.01)	(0.07)	(0.02)	(0.07)	(0.01)	(0.29)
*n* (observations)	252,954	252,912	252,912	252,912	252,912	252,941	252,954
*n* (individuals)	26,717	26,715	26,715	26,715	26,715	26,717	26,717
Pseudo *R* ^2^	0.161	0.166	0.150	0.127	0.106	0.082	0.094

*Note*: Data from the HILDA Survey, from 2003 to 2020. All models are adjusted for a full set of socio‐demographic controls, including state of residence, age group, gender, immigrant, partnership and parenthood and labour‐force status, education, income group, housing tenure, and duration of residence in current address. Standard errors clustered across individuals. Test 2: (β_2020_ − β_2019_)−(β_2019_ − β_2018_) > 0. GCCSA, Greater Capital City Statistical Area; SA, Statistical Area. Statistical significance (two‐sided tests).

^#^ *p* < 0.1.

* *p* < 0.05

** *p* < 0.01.

**Table 2:** Odds ratios (*p* values) from logistic regression models of different reasons for migrating, all changes of address

	Employment reasons	Housing reasons	Amenity reasons	Family reasons	Lifestyle reasons	Involuntary reasons
*Panel 1 (Model 1)*						
Year 2018	1.00	0.96	0.95	0.91	1.01	1.05
	(0.99)	(0.44)	(0.55)	(0.10)	(0.91)	(0.59)
Year 2019 *(reference)*						
**Test 1**: Year 2020	0.78**	0.95	0.83#	0.95	0.74**	0.72**
	(<0.01)	(0.29)	(0.05)	(0.34)	(<0.01)	(<0.01)
**Test 2**: one‐sided *p* value	0.03	0.15	0.07	0.07	0.02	0.04
*n* (observations)	252,954	252,954	252,954	252,954	252,954	252,954
*n* (individuals)	26,717	26,717	26,717	26,717	26,717	26,717
Pseudo *R* ^2^	0.132	0.115	0.068	0.094	0.184	0.030
*Panel 2 (Model 2)*						
Year (continuous)	0.99**	0.99**	1.01*	1.00	1.00	0.98**
	(<0.01)	(<0.01)	(0.03)	(0.17)	(0.75)	(<0.01)
**Test 3**: Year 2020	0.78**	1.00	0.85*	1.01	0.75**	0.76**
	(<0.01)	(0.95)	(0.04)	(0.86)	(<0.01)	(<0.01)
*n* (observations)	252,954	252,954	252,954	252,954	252,954	252,954
*n* (individuals)	26,717	26,717	26,717	26,717	26,717	26,717
Pseudo *R* ^2^	0.132	0.114	0.067	0.093	0.184	0.029

*Note*: Data from the HILDA Survey, from 2003 to 2020. All models are adjusted for a full set of socio‐demographic controls, including state of residence, age group, gender, immigrant, partnership and parenthood and labour‐force status, education, income group, housing tenure, and duration of residence in current address. Standard errors clustered across individuals. Test 2: (β_2020_ − β_2019_) − (β_2019_ − β_2018_) > 0. Statistical significance (two‐sided tests).

^#^ *p* < 0.1.

* *p* < 0.05

** *p* < 0.01.

Panel 1 in Table 1 demonstrates that the odds of migrating in 2020 were statistically significantly lower than in 2019 for address changes (OR = 0.92; *p* < 0.05), as well as migration between SA1s (OR = 0.93; *p* < 0.05) and GCCAs (OR = 0.81; *p* < 0.01). The relevant coefficient was also marginally significant for migration between SA4s (OR = 0.91; *p* < 0.1). When comparing the 2019/2020 rate of change in the odds of migrating to the 2018/2019 rate, we observed statistically lower migration between SA1s (*p* = 0.04) and GCCSAs (*p* = 0.01), and marginally significant differences for address changes (*p* = 0.06). Panel 2 in Table 1 provides more conclusive evidence of a COVID‐disruption to long‐term migration trends, with statistically significant coefficients on the 2020 dummy variables for address changes (OR = 0.94; *p* < 0.05) and migration between SA1s (OR = 0.93; *p* < 0.05), SA3s (OR = 0.93; *p* < 0.05), and GCCCAs (OR = 0.86; *p* < 0.05), and marginally significant coefficients for between SA2s (OR = 0.95; *p* < 0.1) and SA4s (OR = 0.93; *p* < 0.1). Focusing on spatial scales that exhibit statistically significant results for all three tests, we conclude that the odds of migrating internal has declined for changes of address, change of SA1, SA4, and movement between capital cities and regional areas. The magnitude of these shifts, however, appears moderate.

Table 2 presents equivalent evidence for reason‐specific migration. In Panel 1, the 2020 odds of migrating were statistically significantly lower than in 2019 for employment (OR = 0.78; *p* < 0.01), lifestyle (OR = 0.74; *p* < 0.01), and involuntary reasons (0.72; *p* < 0.01). The odds ratio on amenity reasons was also below one and marginally significant (0.83; *p* < 0.1). When comparing the 2019/2020 and 2018/2019 rates of change in the odds of migrating, we observed statistically significant differences for employment (*p* < 0.03), lifestyle (*p* < 0.02), and involuntary (*p* < 0.04) reasons and marginally significant differences for amenity (*p* < 0.1) and family (*p* < 0.1) reasons. Using an alternative specification, Panel 2 in Table 2 confirms the decreases in for the odds of migrating for employment (OR = 0.78; *p* < 0.01), amenity (OR = 0.85; *p* < 0.05), lifestyle (OR = 0.75; *p* < 0.01), and involuntary (OR = 0.76; *p* < 0.01) reasons. Focusing on the reasons that exhibit statistically significant results for all three tests, we conclude that employment and involuntary migration have declined following the onset of the COVID‐19 pandemic. The decline in employment‐related migration aligns with observations in other developed countries (Haslag & Weagley, 2021), and could be explained by a shift to reworking coupled with server lockdowns in 2020. Perhaps more surprising is the decrease in migration due to involuntary reasons, which might result from eviction moratoria for renters put in place for 6 months from April 2020.

### 4.3 Did COVID‐19 alter flows between cities and regional areas?

**Figure 8:**
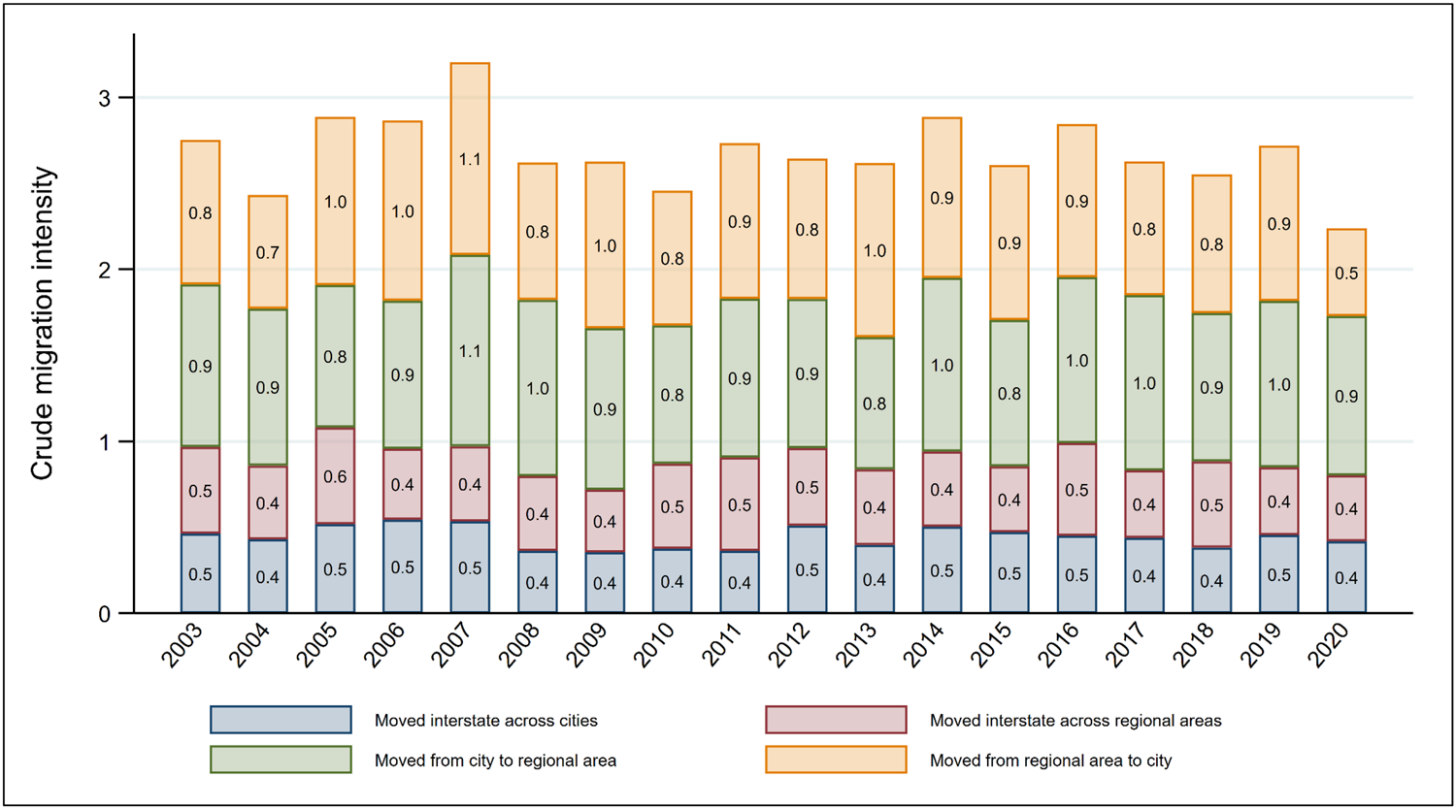
Crude migration intensities between capital cities and rest of state, from 2003 to 2020. Data from the HILDA Survey, from 2003 to 2020. HILDA, Household, Income and Labour Dynamics in Australia.

To gain further insights into how the COVID‐19 pandemic may have shifted migration patterns in Australia, we conduct three sets of additional analyses. First, we explore how the pandemic may have affected migration flows between urban cores and regional areas. To do this, we use a variable characterising the origin and destination area of migrants based on the capital city/rest of state classification. Figure 8 shows changes in the intensity of migration between regional areas and capital cities in 2020 relative to earlier years. Particularly noticeable is the share of people who moved from a regional area to a capital city, which nearly halved between 2019 (0.9%) and 2020 (0.5%). Other flows have remained broadly stable.

We examine this trend more formally by fitting multinomial logistic regression models to this outcome variable and implementing the same three tests described and used before. Abridged results from these models are presented in Table 3 in the form of RRRs. Relative to the baseline category of the outcome variable (i.e., staying within the same capital city), we find strong evidence that the odds of migrating from a regional area to a capital city reduced significantly in 2020. This effect was apparent in each of the three tests and the magnitude of the effect was seemingly large. For example, the RRR on the relevant 2020 dummy variable was 0.57 in Panel 1 (*p* < 0.01) and 0.62 in Panel 2 (*p* < 0.01). The test assessing the annual rate of change in the odds of leaving a regional area is also highly statistically significant (*p* < 0.01). Overall, these results suggest that the COVID‐19 pandemic may have precluded movement from regional areas towards urban cores, but did not result in a statistically significant increase in moves from capital cities to regional areas. This national‐level trend is however the result of state‐specific changes in different directions, as highlighted in Section 2.

**Table 3:** Relative risk ratios (*p* values) from multinomial logistic regression models of migration between capital cities and regional areas (baseline: Stayed in or moved within a capital city)

	Moved interstate to different city	Stayed in same regional area	Moved interstate to a different regional area	Moved from capital city to regional area	Moved from regional area to capital city
*Panel 1 (Model 1)*					
Year 2018	0.84	0.99	1.25	0.88	0.88
	(0.30)	(0.22)	(0.16)	(0.25)	(0.29)
Year 2019 *(reference)*					
**Test 1**: Year 2020	0.89	1.02*	0.97	0.95	0.57**
	(0.47)	(0.03)	(0.87)	(0.65)	(<0.01)
**Test 2**: one‐sided *p* value	0.15	0.25	0.25	0.18	<0.01
*n* (observations)	252,941				
*n* (individuals)	26,717				
Pseudo *R* ^2^	0.068				
*Panel 2 (Model 2)*					
Year (continuous)	0.99*	1.01**	1.00	1.00	1.00
	(0.05)	(<0.01)	(0.73)	(0.64)	(0.45)
**Test 3**: Year 2020	0.99	1.01	0.89	1.02	0.62**
	(0.93)	(0.18)	(0.40)	(0.84)	(<0.01)
*n* (observations)	252,941				
*n* (individuals)	26,717				
Pseudo *R* ^2^	0.067				

*Note*: Data from the HILDA Survey, from 2003 to 2020. All models are adjusted for a full set of socio‐demographic controls, including age group, gender, immigrant, partnership and parenthood and labour‐force status, education, income group, housing tenure, and duration of residence in current address. This model cannot be adjusted for state of residence due to collinearity with the outcome variable. Standard errors clustered across individuals. Test 2: (β_2020_ − β_2019_) − (β_2019_ − β_2018_) > 0. Statistical significance (two‐sided tests).

^#^ *p* < 0.1.

* *p* < 0.05

** *p* < 0.01.

### 4.4 Did COVID‐19 change the realisation of migration intentions?

**Figure 9:**
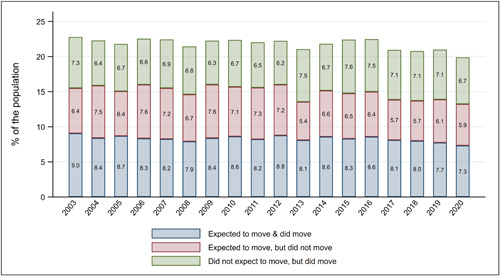
Migration intentions and their realisations, from 2003 to 2020. Data from the HILDA Survey, from 2003 to 2020. The fourth category ‘Did not expect to move and did not move' was not included in the graph because it accounts for over 75% of the population, which would make it difficult to visualise trends in the other categories. HILDA, Household, Income and Labour Dynamics in Australia.

We now examine whether the pandemic has shifted the formation and realisation of migration intentions. Figure 9 shows small, yet noticeable decreases in the share of people who ‘expected to move, but did not move’ (7.7% in 2019 and 7.3% in 2020) and people who ‘did not expect to move, but moved’ (7.1% in 2019 and 6.7% in 2020). These decreases occurred at the expense of increases in the share of people who ‘did not expect to move and did not move’ (omitted from the figure), which grew from 79.1% in 2019 to 80.1% in 2020.

To examine these trends more formally, we apply the same analytic strategy used in the previous section, fitting a multinomial logistic regression model on an outcome variable capturing the match between individuals' self‐reported likelihood to move and their subsequent address changes (Table 4). Relative to the baseline category of the outcome variable (i.e., not expecting to move and not moving), we find that the onset of the pandemic reduced the likelihood of people realising their migration intentions (in two of three tests conducted), undertaking unexpected moves (in two of three tests), and those failing to realise move expectations (in just one of three tests). We take these results as indicating that the COVID‐19 pandemic generated a degree of unpredictability that made both unexpected moves more likely and expected moves less likely.

### 4.5 Drivers of migration

We conclude the analysis by exploring possible changes in the socio‐demographic factors that facilitate or constrain migrations. To test this proposition, we fitted a series of logistic regression models—at each spatial scale—using HILDA Survey data from years 2019 and 2020. Because of the dimensions and complexity of the regression table, we present the tabular results in Appendix Table A3 and summarise the key patterns here.

The coefficients on all of the control variables exhibit the expected sign. The odds of migrating decrease with age, home ownership and duration of residence, and increase with education. Further, the results paint a picture of temporal stability in the predictors of migration at different spatial scales, as evidenced by a preponderance of statistically nonsignificant ORs in the interaction effects. For example, we observe no changes in the age selectivity of migration at all spatial scales or in the role of socioeconomic factors such as employment status and home ownership. There are, however, a few noticeable exceptions. In particular, tertiary‐educated individuals were less likely to migrate between SA4s—which broadly corresponds to labour‐market areas—and between capital cities and rest of state in 2020 than in 2019. The deterring effect of duration of residence, which is often used as a proxy for place attachment, also increased in 2020 relative to 2019, particularly for residential mobility and interstate migration. The latter suggests that the weight of local ties is greater in adverse circumstances. Overall, we conclude that the pandemic did not dramatically shift the socio‐demographic drivers of internal migration in Australia.

## 5 DISCUSSION AND CONCLUSION

This study has added to the nascent literature on post‐COVID‐19 patterns of internal migration in developed countries by coupling descriptive analysis of aggregate migration count and flow data from the Australian Bureau of Statistics with regression‐based analysis of long‐term microdata from the HILDA Survey. The latter enabled us to control for possible changes in population composition that may confound internal migration trends, extend the evidence to lower levels of geography, and explore reasons for migrating and the realisation of migration intentions.

As for other OECD countries, Australia recorded a decline in the intensity of migration, with interstate migration decreasing by 8.75% in 12 months to December 2020, reaching the lowest level on record at 1.44%. While modest, this decrease is significant because it occurred at the back of a 30‐year decline in the intensity of internal migration (Kalemba et al., 2022). However, the rebound in the intensity of interstate migration in 2021 suggests that the impact of COVID‐19 is likely to be short‐lived and less pronounced than in previous recessions. These results contradict early expert opinion surveys, which predicted a 30% decline in interstate migration in Australia (Bernard et al., 2020). The lower than anticipated decline in interstate migration may be the result of the generous income‐support measures put in place by the Australian Government from April 2020 to March 2021, which likely cushioned the economic fallout of the early phases of the pandemic.

Our analyses of the HILDA Survey suggest that, in 2020, the decline extended to residential mobility and migration between capital cities and regional areas. It is worth noting that the results are sensitive to the selection of comparison method, which highlights the importance of stringent methodological choices. More importantly, the decline in residential mobility recorded in 2020 was partly driven by a reduction in involuntary migration. This is most likely linked to the 6‐month eviction moratorium that ended in September 2020. This suggests that residential mobility for involuntary reasons is likely to have rebounded in 2021.

More importantly, we found a decline in employment‐related migration. Specifically, our analyses revealed a statistically significant decline in the incidence of migration due to employment motives, leading to the lowest level on record, with less than 1.8% of the Australian population moving for such purposes in 2020. The downward trend in employment‐related migration precedes COVID‐19. This trend has been explained by a general decline in the dynamism of the labour market with fewer people changing jobs (Kalemba et al., 2022; Molloy et al., 2014). Post‐COVID‐19, the decline in employment‐related migration has been attributed to an increase in teleworking among white‐collar workers (Haslag & Weagley, 2021). While we found tertiary‐educated individuals to be less likely to migrate between SA4s and capital cities and rest of state in 2020 than in 2019, the other determinants of migration remained broadly stable. The only noticeable exception was duration of residence, whose restraining effect increased at all spatial scales. This suggests that individuals became more rooted and placed greater value on local ties when navigating the adverse circumstances caused by the COVID‐19 pandemic. This situation is likely to have reversed after restrictions and lockdowns eased, which could have contributed to the small rebound in interstate migration observed in 2021.

**Table 4:** Relative risk ratios (*p* values) from multinomial logistic regression models of migration behaviour relative to expectations (baseline: Did not expect to move and did not move)

	Expected to move and moved	Expected to move, but did not move	Did not expect to move, but moved
*Panel 1 (Model 1)*			
Year 2018	1.02	0.93#	0.98
	(0.58)	(0.08)	(0.65)
Year 2019 *(reference)*			
**Test 1**: Year 2020	0.92*	0.93	0.91*
	(0.05)	(0.10)	(0.03)
**Test 2**: one‐sided *p* value	0.20	0.02	0.07
*n* (observations)	252,870		
*n* (individuals)	26,716		
Pseudo *R* ^2^	0.130		
*Panel 2 (Model 2)*			
Year (continuous)	0.98**	0.98**	1.00
	(<0.01)	(<0.01)	(0.26)
**Test 3**: Year 2020	0.92*	0.99	0.92*
	(0.02)	(0.87)	(0.02)
*n* (observations)	252,870		
*n* (individuals)	26,716		
Pseudo *R* ^2^	0.130		

*Note*: Data from the HILDA Survey, from 2003 to 2020. All models are adjusted for a full set of socio‐demographic controls, including state of residence, age group, gender, immigrant, partnership and parenthood and labour‐force status, education, income group, housing tenure, and duration of residence in current address. Standard errors clustered across individuals. Test 2: (β_2020_ − β_2019_)−(β_2019_ − β_2018_) > 0. Statistical significance (two‐sided tests).

^#^ *p* < 0.1.

* *p* < 0.05

** *p* < 0.01.

The fact that the observed decline in internal migration held once socio‐demographic characteristics were controlled for further suggests that it is not the product of a changing population composition. Rather, it may be attributed to behavioural shifts. A noticeable change has been a statistically significant increase in the odds of individuals staying in regional areas, more so than a growth in the movement out of cities. This change in the spatial patterning of migration requires further research. While providing a wealth of individual‐level information, the HILDA Survey is not optimally suited for the analysis of detailed migration flows. The forthcoming release of the 2021 Australian Census data, which was fielded in August 2021, will provide a unique opportunity to explore the spatial impact of COVID‐19 in more depth through origin–destination matrices. Indeed, it may take a few more years for some of the effects of the COVID‐19 pandemic on internal migration to emerge, as structural changes stemming from the pandemic pick up pace. For example, shifts in work patterns, particularly teleworking, are becoming increasingly accepted, and this may progressively transform the level, direction, and composition of internal migration in ways that may not yet be apparent.

In the meantime, the evidence assembled in this paper demonstrates that the COVID‐19 pandemic has exacerbated a long‐term trend toward reduced population movement. There is considerable debate, in Australia and other developed countries, as to whether people are increasingly ‘stuck’ or ‘rooted’ (Foster, 2016) and whether this trend is a problem for the functioning of the labour market and other social institutions. The small rebound of migration in 2021 suggests that after 30 years of a decline in internal migration, the intensity of interstate migration may have ‘bottomed out’. Perhaps it cannot possibly go any lower.

## Data Availability

The data that support the findings of this study are available on request from the corresponding author.

## Footnotes

1 Results for regional areas are not reported here because they are simply the reverse.

2 Across all internal‐migration measures based on changes across administrative areas, the 2002 data displayed a sharp increase in internal migration followed by a sharp decline. Unlike other HILDA Survey waves, these patterns are inconsistent with those emerging in other Australian data sources. Further, the patterns are not apparent in self‐reported measures of migration.

3 The term ‘migration intensity’ encompasses both migration rates and probabilities (Van Imhoff & Keliman, 1991).

## APPENDIX A

### A.1

See Tables A1–A3.

**Table A1:** Geographical classifications used to measure internal migration

Spatial scale	Definition	# units
Statistical Areas Level 1 (SA1)	SA1s generally have a population of 200–800 people, and an average population of about 400 people. SA1s are designed to be either urban or rural in character and to represent Aboriginal and Torres Strait Islander communities as accurately as possible, particularly in remote areas. SA1s are generally internally connected by road transport.	61,845
SA2	SA2s generally have a population between 3000 and 25,000 with an average of about 10,000 people. Their purpose is to represent a community that interacts together socially and economically.	2473
SA3	SA3s are designed to have populations between 30,000 and 130,000 people. SA3s are often the functional areas of regional towns and cities with a population in excess of 20,000 or clusters of related suburbs around urban commercial and transport hubs within the major urban areas.	359
SA4	SA4s are the largest substate regions in the Main Structure of the ASGS Most SA4s have a population above 100,000 people to provide sufficient sample size for Labour Force estimate. SA4s are designed to incorporate both labour supply (where people live) and demand (where people work).	108
Greater Capital Cities Statistical Areas (GCCSA)	Each state and territory is divided in greater capital city and rest of state, with the expectation of the Australian Capital Territory and other territories.	16
States and territories	New South Wales, Victoria, Queensland, South Australia, Western Australia, Tasmania, Northern Territory, Australian Capital Territory and other territories (Jervis Bay Territory, Territory of Christmas Island, Territory of the Cocos (Keeling) Islands and Norfolk Island).	9

*Note*: Sourced from Australian Bureau of Statistics, Main Structure and Greater Capital Statistical Areas (Main Structure and Greater Capital City Statistical Areas. Australian Bureau of Statistics (abs.gov.au). Excludes special purpose codes locate outside of Australia.

**Table A2:** Descriptive statistics on analytic variables

	%	Standard error of the mean
**Outcome variables** (time *t*)		
*Move type*		
Changed address since last interview	16.2	0.072
Changed SA1 since last interview	15.4	0.071
Changed SA2 since last interview	12.2	0.064
Changed SA3 since last interview	8.5	0.055
Changed SA4 since last interview	6.0	0.047
Changed GCCSA since last interview	2.7	0.032
Changed state since last interview	1.5	0.024
*Move reason*		
Employment reasons	2.4	0.030
Housing reasons	6.3	0.047
Amenity reasons	1.4	0.022
Family reasons	4.1	0.038
Involuntary reasons	1.9	0.027
Lifestyle reasons	1.8	0.025
*Move expectations*		
Expected to move (at *t* − 1) and did move (by *t*)	8.3	0.055
Expected to move (at *t* − 1), but did not move (by *t*)	6.6	0.049
Did not expect to move (at *t* − 1), but moved (by *t*)	6.9	0.050
Did not expect to move (at *t* − 1) and did not move (by *t*)	78.3	0.082
*Urban‐rural mobility*		
Stayed in same city	58.1	0.097
Moved interstate across cities	0.4	0.013
Stayed in same regional area	39.2	0.096
Moved interstate across regional areas	0.4	0.013
Moved from city to regional area	0.9	0.019
Moved from regional area to city	0.9	0.018
**Explanatory variables** (time *t* − 1)		
State/territory of residence		
New South Wales	29.9	0.090
Victoria	25.0	0.085
Queensland	20.9	0.080
South Australia	9.2	0.057
Western Australia	9.1	0.057
Tasmania	3.2	0.034
Northern Territory	0.7	0.017
Australian Capital Territory	2.0	0.027
Age group		
15–24 years	18.6	0.076
25–44 years	34.0	0.093
45–64 years	30.7	0.090
65+ years	16.7	0.073
Respondent is female	52.7	0.098
Respondent is immigrant	21.2	0.081
Partnership status		
Married/cohabitating	63.0	0.096
Divorced, separated, widowed	13.7	0.068
Never married	23.3	0.084
Respondent has children	66.0	0.094
Respondent has University‐level qualifications	23.4	0.084
Labour‐force status		
Employed full‐time	42.6	0.098
Employed part‐time	21.1	0.081
Unemployed	3.7	0.037
Not in the labour force	32.6	0.093
Household income		
Bottom quartile	25.0	0.085
Middle two quartiles	50.0	0.098
Top quartile	25.0	0.085
Housing tenure		
Owns home	69.1	0.091
Rents	28.1	0.088
Other arrangement	2.7	0.032
Duration of residence in current address		
Less than 1 year	15.9	0.072
1–5 years	29.4	0.089
5–10 years	17.1	0.074
10+ years	34.5	0.093
Insufficient information	3.1	0.034

*Note*: Data from the HILDA Survey, 2003–2020. Observations pooled across all survey waves. SA: Statistical Area. GCCSA: Greater Capital City Statistical Area.

**Table A3:** Odds ratios (*p* values) from logistic regression models of selected mobility outcomes (2019 vs. 2020)

	Changed address	Changed SA1	Changed SA2	Changed SA3	Changed SA4	Changed GCCSA	Changed state
***Main effects***							
Year 2020	0.77	0.77	0.76	0.70	0.98	1.34	0.81
Age group							
15–24 years *(reference)*							
25–44 years	0.73**	0.70**	0.79**	0.88	1.02	0.99	1.10
45–64 years	0.42**	0.39**	0.44**	0.49**	0.57**	0.51**	0.63#
65+ years	0.33**	0.30**	0.31**	0.34**	0.40**	0.31**	0.44*
Respondent is female	1.03	1.01	1.00	1.00	1.00	1.04	1.00
Partnership status	0.98	0.98	1.03	1.01	0.99	0.89	0.87
Married/cohabitating *(reference)*							
Divorced, separated, widowed	1.06	1.08	1.07	1.34**	1.41**	1.37#	1.41
Never married	0.93	0.95	0.99	1.08	1.10	1.23	1.15
Respondent has children	0.91	0.89#	0.77**	0.69**	0.66**	0.76*	0.64**
Respondent has University qualifications	1.08	1.08	1.22**	1.27**	1.52**	1.36**	1.45*
Labour‐force status							
Employed full‐time *(reference)*							
Employed part‐time	0.78**	0.81**	0.86*	0.86#	0.90	1.07	1.12
Unemployed	0.98	0.98	0.84	0.84	0.93	1.00	1.10
Not in the labour force	0.84*	0.83*	0.83*	0.88	1.04	1.30#	1.32
Household income							
Bottom income quartile *(reference)*							
Middle two income quartiles	0.97	0.92	0.86*	0.90	0.94	0.92	0.89
Top income quartile	1.07	1.01	0.97	1.10	1.14	1.08	1.10
Housing tenure							
Owns home *(reference)*							
Rents	4.11**	3.98**	3.24**	2.83**	2.54**	1.90**	1.85**
Other arrangement	3.61**	3.31**	2.97**	3.21**	3.01**	2.52**	1.86#
Duration of residence in current address							
Less than 1 year *(reference)*							
1–5 years	0.57**	0.55**	0.61**	0.57**	0.62**	0.66**	0.52**
5–10 years	0.42**	0.41**	0.42**	0.38**	0.40**	0.40**	0.30**
10 or more years	0.33**	0.33**	0.37**	0.36**	0.39**	0.38**	0.19**
***Interaction effects***							
Year 2020 × …							
25–44 years	1.03	1.05	1.09	1.09	0.93	0.85	0.82
45–64 years	1.08	1.14	1.15	1.20	1.12	1.27	0.95
65+ years	1.13	1.20	1.47#	1.44	1.40	1.59	1.14
Respondent is female	1.06	1.07	1.07	1.09	1.06	1.00	1.06
Respondent is immigrant	0.97	0.95	0.91	1.08	1.02	0.86	0.88
Divorced, separated, widowed	1.07	0.99	1.07	0.90	0.82	0.84	0.67
Never married	1.14	1.14	1.15	1.12	1.19	1.05	1.15
Respondent has children	0.92	0.90	0.97	1.03	1.13	1.13	1.60#
Respondent has University qualifications	0.95	0.98	0.90	0.92	0.77*	0.72#	0.68
Employed part‐time	1.20*	1.16	1.11	1.06	0.93	0.75	0.82
Unemployed	1.01	1.01	1.27	1.50*	1.25	1.33	1.14
Not in the labour force	0.90	0.90	0.89	0.94	0.81	0.71	0.78
Middle two income quartiles	0.92	0.90	0.90	0.86	0.80	0.61*	0.60*
Top income quartile	0.97	0.97	0.98	0.85	0.79	0.53**	0.53*
Rents home	0.98	1.03	1.10	1.05	1.03	1.02	0.94
Other housing arrangement	0.96	1.05	1.18	1.04	1.02	0.87	1.53
1–5 years in current address	1.29**	1.27**	1.13	1.27*	1.01	1.07	1.61*
5–10 years in current address	1.07	1.07	1.00	1.26	1.13	1.15	1.29
10+ years in current address	1.54**	1.46**	1.26#	1.23	1.07	1.00	2.05#
*n* (observations)	32,465	32,443	32,443	32,443	32,443	32,358	32,360
*n* (individuals)	17,177	17,167	17,167	17,167	17,167	17,157	17,157
Pseudo *R* ^2^	0.162	0.168	0.148	0.129	0.110	0.089	0.105

*Note*: Data from the HILDA Survey, 2019–2020. All models are also adjusted for state/territory fixed effects and their interactions with survey year. Standard errors clustered across individuals. Statistical significance (two‐sided tests).

^#^ *p* < 0.1.

* *p* < 0.05

** *p* < 0.01.

## References

[psp2626-bib-0001] Aassve, A. , Cavalli, N. , Mencarini, L. , Plach, S. , & Livi Bacci, M. (2020). The COVID‐19 pandemic and human fertility. Science, 369(6502), 370–371. 32703862 10.1126/science.abc9520

[psp2626-bib-0002] ABS . (2020). Household impacts of COVID‐19 survey.

[psp2626-bib-0003] ABS . (2021). Australian fertility rate hits record low.

[psp2626-bib-0004] Alvarez, M. , Bernard, A. , & Lieske, S. N. (2021). Understanding internal migration trends in OECD countries. Population, Space and Place, 27, e2451.

[psp2626-bib-0005] Anderson, B. , Poeschel, F. , & Ruhs, M. (2021). Rethinking labour migration: Covid‐19, essential work, and systemic resilience. Comparative Migration Studies, 9(1), 45. 34608433 10.1186/s40878-021-00252-2 PMC8480999

[psp2626-bib-0006] Argent, N. , & Plummer, P. (2022). Counter‐urbanisation in pre‐pandemic times: Disentangling the influences of amenity and disamenity. Australian Geographer, 1–25. 10.1080/00049182.2022.2043807

[psp2626-bib-0007] Argent, N. , Tonts, M. , Jones, R. , & Holmes, J. (2010). Amenity‐led migration in rural Australia: A new driver of local demographic and environmental change? Demographic change in Australia's rural landscapes (pp. 23–44). Springer.

[psp2626-bib-0008] Bell, M. , Charles‐Edwards, E. , Bernard, A. , & Ueffing, P. (2018). Global trends in internal migration. In T. Champion , T. Cooke , & I. Shuttleworth (Eds.), Internal migration in the developed world: Are we becoming less mobile? (pp. 76–97). Routledge.

[psp2626-bib-0009] Bell, M. , Charles‐Edwards, E. , Ueffing, P. , Stillwell, J. , Kupiszewski, M. , Kupiszewska, D. J. P. , & Review, D. (2015). Internal migration and development: Comparing migration intensities around the world. Population and Development Review, 41(1), 33–58.

[psp2626-bib-0010] Bell, M. , & Muhidin, S. (2009). *Cross‐national comparison of internal migration*. Human Development Research Paper (HDRP) Series, vol. 30.

[psp2626-bib-0011] Bernard, A. , Bell, M. , & Charles‐Edwards, E. (2014). Life‐course transitions and the age profile of internal migration. Population and Development Review, 40(2), 213–239.

[psp2626-bib-0012] Bernard, A. , Bell, M. , & Charles‐Edwards, E. (2016). Internal migration age patterns and the transition to adulthood: Australia and Great Britain compared. Journal of Population Research, 33(2), 123–146.

[psp2626-bib-0013] Bernard, A. , Charles‐Edwards, E. , Alvarez, M. , Wohland, P. , Loginova, J. , & Kalemba, S. (2020). *Anticipating the impact of COVID‐19 on internal migration*.

[psp2626-bib-0014] Berrington, A. , Ellison, J. , Kuang, B. , Vasireddy, S. , & Kulu, H. (2022). Scenario‐based fertility projections incorporating impacts of COVID‐19. Population, Space and Place, 28(2), e2546.

[psp2626-bib-0015] Bick, A. , Blandin, A. , & Mertens, K. (2021). *Work from home before and after the Covid‐19 outbreak*. Available at SSRN 3786142.

[psp2626-bib-0016] Bonifazi, C. , Heins, F. , Licari, F. , & Tucci, E. (2020). The regional dynamics of internal migration intensities in Italy. Population, Space and Place, 27(7), e2331.

[psp2626-bib-0017] Borsellino, R. , Bernard, A. , Charles‐Edwards, E. , & Corcoran, J. (2022). A regional renaissance? The shifting geography of internal migration under COVID‐19. Australian Geographer, 1–19.

[psp2626-bib-0018] Campbell, P. (2019). Dispositional traits and internal migration: Personality as a predictor of migration in Australia. Journal of Research in Personality, 78, 262–267.

[psp2626-bib-0019] Charles‐Edwards, E. , Wilson, T. , Bernard, A. , & Wohland, P. (2021). How will COVID‐19 impact Australia's future population? A scenario approach. Applied Geography, 134, 102506. 36536836 10.1016/j.apgeog.2021.102506 PMC9753126

[psp2626-bib-0020] Clark, W. A. V. , & Lisowski, W. (2018). Examining the life course sequence of intending to move and moving. Population, Space and Place, 24(3), e2100. 29731698 10.1002/psp.2100 PMC5932626

[psp2626-bib-0021] Clark, W. A. V. , & Lisowski, W. (2019). Extending the human capital model of migration: The role of risk, place, and social capital in the migration decision. Population, Space and Place, 25(4), e2225.

[psp2626-bib-0022] Coulter, R. , Van Ham, M. , & Feijten, P. (2011). A longitudinal analysis of moving desires, expectations and actual moving behaviour. Environment and Planning A: Economy and Space, 43(11), 2742–2760.

[psp2626-bib-0023] Courgeau, D. , Muhidin, S. , & Bell, M. (2012). Estimating changes of residence for cross‐national comparison. Population (English Edition), 67(4), 631–651.

[psp2626-bib-0024] CPoP . (2022). 2022–23 budget: Australia's future population. Australian Government.

[psp2626-bib-0025] Crown, D. , Gheasi, M. , & Faggian, A. (2020). Interregional mobility and the personality traits of migrants. Papers in Regional Science, 99(4), 899–914.

[psp2626-bib-0026] Fielding, T. , & Ishikawa, Y. (2021). COVID‐19 and migration: A research note on the effects of COVID‐19 on internal migration rates and patterns in Japan. Population, Space and Place, 27(6), e2499. 34512211 10.1002/psp.2499 PMC8420226

[psp2626-bib-0027] Ford, T. (1999). Understanding population growth in the peri‐urban region. International Journal of Population Geography, 5(4), 297–311. 12322307 10.1002/(SICI)1099-1220(199907/08)5:4<297::AID-IJPG152>3.0.CO;2-O

[psp2626-bib-0028] Foster, T. (2016). *Rooted or stuck? The causes and consequences of American mobility decline* (Doctoral dissertation). University of Washington.

[psp2626-bib-0029] Fostik, A. (2021). COVID‐19 and fertility in Canada: A commentary. Canadian Studies in Population, 48(2), 217–224. 34493896 10.1007/s42650-021-00054-y PMC8414452

[psp2626-bib-0030] Van Der Gaag, N. , & Van Wissen, L. (2008). Economic determinants of internal migration rates: A comparison across five European countries. Tijdschrift Voor Economische en Sociale Geografie, 99(2), 209–222.

[psp2626-bib-0031] Gamlen, A. (2020). Migration and mobility after the 2020 pandemic: The end of an age. IOM's Migration Research High Level Advisers, 2–14.

[psp2626-bib-0032] Gillespie, B. J. , Mulder, C. H. , & Eggleston, C. M. (2021). Measuring migration motives with open‐ended survey data: Methodological and conceptual issues. Population, Space and Place, 27, e2448. 34594163 10.1002/psp.2448 PMC8459254

[psp2626-bib-0033] González‐Leonardo, M. , López‐Gay, A. , Newsham, N. , Recaño, J. , & Rowe, F. (2022). *Understanding patterns of internal migration during the COVID‐19 pandemic in Spain*. 10.1002/psp.2578 PMC9350359 35942493

[psp2626-bib-0034] Haslag, P. H. , & Weagley, D. (2021). *From LA to Boise: How migration has changed during the COVID‐19 pandemic*. Available at SSRN 3808326.

[psp2626-bib-0035] IOM . (2022). DTM (COVID‐19) global mobility restrictions overview . 9 May 2022.

[psp2626-bib-0036] Kalemba, S. V. , Bernard, A. , Charles‐Edwards, E. , & Corcoran, J. (2020). Decline in internal migration levels in Australia: Compositional or behavioural effect? Population, Space and Place, 27(7), e2341.

[psp2626-bib-0037] Kalemba, S. V. , Bernard, A. , Corcoran, J. , & Charles‐Edwards, E. (2022). Has the decline in the intensity of internal migration been accompanied by changes in reasons for migration? Journal of Population Research, 39, 279–313.

[psp2626-bib-0038] Korpi, M. , & Clark, W. A. V. (2017). Human capital theory and internal migration: Do average outcomes distort our view of migrant motives? Migration Letters, 14(2), 237–250. 28936225 PMC5604464

[psp2626-bib-0039] Long, L. (1991). Residential mobility differences among developed countries. International Regional Science Review, 14(2), 133–147. 12284725 10.1177/016001769101400202

[psp2626-bib-0040] Luppi, F. , Arpino, B. , & Rosina, A. (2020). The impact of COVID‐19 on fertility plans in Italy, Germany, France, Spain, and the United Kingdom. Demographic Research, 43, 1399–1412.

[psp2626-bib-0041] Molloy, R. , Smith, C. L. , & Wozniak, A. K. (2014). Declining migration within the US: The role of the labor market. National Bureau of Economic Research.

[psp2626-bib-0042] Monras, J. (2018). *Economic shocks and internal migration*.

[psp2626-bib-0043] Morosow, K. , & Kolk, M. (2020). How does birth order and number of siblings affect fertility? A within‐family comparison using Swedish register data. European Journal of Population, 36(2), 197–233. 32256257 10.1007/s10680-019-09525-0 PMC7113329

[psp2626-bib-0044] Newland, K. (2020). Will international migration governance survive the COVID‐19 pandemic. Migration Policy Institute.

[psp2626-bib-0045] OECD . (2021). International Migration Outlook 2021. OECD.

[psp2626-bib-0046] Rees, P. , Bell, M. , Kupiszewski, M. , Kupiszewska, D. , Ueffing, P. , Bernard, A. , Charles‐Edwards, E. , & Stillwell, J. (2017). The impact of internal migration on population redistribution: An international comparison. Population, Space and Place, 23(6), e2036.

[psp2626-bib-0047] Rogers, A. , & Castro, L. J. (1981). Model migration schedules. International Institute for Applied System Analysis (IIASA).

[psp2626-bib-0048] Sander, N. , & Bell, M. (2014). Migration and retirement in the life course: An event history approach. Journal of Population Research, 31(1), 1–27.

[psp2626-bib-0049] Stawarz, N. , Rosenbaum‐Feldbrügge, M. , Sander, N. , Sulak, H. , & Knobloch, V. (2022). The impact of the COVID‐19 pandemic on internal migration in Germany: A descriptive analysis. Population, Space and Place, 28, e66. 10.1002/psp.2566 PMC9110994 35601664

[psp2626-bib-0050] Summerfield, M. , Garrard, B. , Jim, Y. , Kamath, R. , Macalad, N. , Watson, N. , Wilkins, R. , & Wooden, M. (2021). HILDA user manual release 20. Applied Economic and Social Research, University of Melbourne.

[psp2626-bib-0051] Thomas, M. , Gillespie, B. , & Lomax, N. (2019). Variations in migration motives over distance. Demographic Research, 40, 1097–1110.

[psp2626-bib-0052] Vidal, S. , Perales, F. , Lersch, P. M. , & Brandén, M. (2017). Family migration in a cross‐national perspective: The importance of institutional and cultural context. Demographic Research, 36, 307–338.

[psp2626-bib-0053] Watson, N. (2020). Measuring geographic mobility: Comparison of estimates from longitudinal and cross‐sectional data. Survey Research Methods, 14(1), 1–18.

[psp2626-bib-0054] Watson, N. , & Wooden, M. P. (2012). The HILDA survey: A case study in the design and development of a successful household panel survey. Longitudinal and Life Course Studies, 3(3), 369–381.

[psp2626-bib-0055] Wilson, T. , & Grossman, I. (2021). How has COVID‐19 affected Australia's internal migration trends and patterns? Interaction, 49(4), 17–20.

